# Thoughts on Ill-Health

**Published:** 1890-08-09

**Authors:** 


### THOUGHTS ON ILL-HEALTH.

### I.—NOT QUITE "FIT."

We are no great admirers of the slang of the present day, but there is no denying that some of its words and phrases fill up distinct gaps in our language, and have considerable force and picturesqueness, when rightly used. And pre-eminent among these words is, we think, that which appears in our heading. "I'm quite fit again, thanks." How exactly the word expresses the state of health which makes everything a pleasure, in which work and play, pleasures, aye, and even anxieties, difficulties, and perplexities can be lightly and cheerfully borne. Cheerfully—why not? We are "fit" for them. Man was not made to live in ease and idleness; he is never so much himself as when breasting the waves, clearing away the brushwood which impedes his progress, forcing the earth to yield to him of her store: if only, ah! if only, he is fit, *i.e.*, has health and strength, for the task.

What of those—and we fear that there are many such—who seem doomed to be never quite fit for life? True, the ill-health of many persons is directly traceable to carelessness, mismanagement, self-indulgence, or idleness, and may therefore be cured; but, leaving all these good people out of account, we fear there are still many whose constitutions are radically defective, who may be patched up now and again by their doctors, but who for ever remain what our country folk expressively call "poor things,"—who never feel, except perhaps at rare intervals, really "fit" for life. It is well for those who enjoy good health to reflect for once in a way on the lot of those who are less fortunate. Never to feel quite fit for life, with its various vicissitudes—what does that mean but a constant struggle, a perpetual sense of the burden and strain of daily life, a weariness which makes troubles doubly hard, and too often turns even pleasures into pains? There is nothing very serious the matter with these people; sometimes they are almost tempted to wish there were, for then they would be recognised as invalids, and the every-day life for which they feel unfit would be exchanged for a more appropriate, if a less inviting, one. They are only "in weak health," or "not strong"—in other words, "not fit." Not for them are the "wild joys of living"; the effervescence of life is gone, and all that it can offer oftentimes seems to them "weary, stale, flat, and unprofitable." That odious "bodily presence" of theirs, with its aches and pains, its discomforts and its needs, makes itself felt when it is least wanted, spoiling a bit of work, breaking the much-needed rest, dashing the bright, careless spirits which a walk on the breezy moorland, or a game with some happy, rollicking children, may induce in them for a few brief moments. The man who is strong and well is so used to feeling himself equal to life that he cannot understand this perpetual and most trying sense of unfitness; or if he does get a glimmering of it, he is apt to class the man of weak health and the man of weak intellect together, to feel the same sort of condescending pity for one who is handicapped by his ailments, as for poor Mr. Tulliver, with his pathetic lament—"The world's too many for me."

This sort of pity is rather a bitter pill to swallow, for one who feels that he has powers within him which might lead to great results, if only he were not hampered at every turn; still, he has not perhaps much right to complain; for, as things go, the world must needs judge of men by what they do—not by what they might have done. But is this condescending pity, or this rather callous judgment by results, all that he has to put up with? Are not some of us apt to indulge in a certain resentment against one who never seems fit for what he has to do,—to feel vexed and impatient when we hear day after day that the same headache goes on, when we see by the tired eyes, or the dragging gait, that the same old answer will come once more—"Not very well to-day, thank you."

"I can't bear to see people ill; it makes me feel sometimes as if I loathed them. I hate the sight of weakness!" confessed a lady not long ago—a lady who was nevertheless well known for her charities; and if there are few amongst us who would so candidly own to such a feeling, there are probably many who can to a certain extent understand it.

Sometimes we work off our vexation by mocking at the sufferer a little—"Headache again? Oh yes, *of course!* When haven't you a headache?" Sometimes we feel too injured even to make a joke of it, and turn away from the pinched lips and drooping form with strong distaste, as we might turn from the skeleton at a feast.

Rather brutal it sounds—does it not?—when put down in black and white. Well, and perhaps that is just what it is. Everybody knows how, when a beast or bird is sickly, or wounded, its fellows will often set upon the poor creature, pecking at or ill-treating it as though it were an enemy, instead of a friend in distress. We do not call them cruel; we believe that their instinct teaches them that health is the proper condition of the body, and that ill-health arouses in them an instinctive repugnance, impelling them to treat their comrades with seeming cruelty, but perhaps with what is in the long run the truest kindness. Possibly the abandonment by the savages of their aged parents is, at least in part, the result of this instinct: and may it not be that the feeling of repulsion which creeps over some of us as we look at a sickly person is the immediate outcome of a healthy instinct—of a sense that here is something abnormal, something unnatural, something which ought not to be?

But while we share our instincts with the brutes, we are endowed with other and finer qualities, which must control, check, and sometimes over-ride these instincts. Our sense of justice, if not our compassion, should surely prevent the healthy repugnance with which we regard ill-health from blossoming into any feeling of dislike for the unfortunate victim of that ill-health.

We have already touched upon the depression and sense of defeat with which those who are handicapped by ill-health think over the career which might have been theirs, the fame which they might have won, if only their minds had been helped, instead of hindered, by their bodies. Even if they are without much ambition or aspiration, they must at least care that their own lives should be full, interesting, and enjoyable; and how trying it must be to a person of any activity of mind to find himself obliged to give up one pursuit after another, to dock off, here a bit of work, there a favourite amusement, to settle down to the humdrum and monotonous life which his health demands—and then perhaps to find that he can only just keep himself going, like a rusty old bit of clockwork which the slightest jar may upset!

Very trying, again, is the sense which so often oppresses a person who is never really well, that in spite of all his efforts to prevent it, his character is losing in force, in individuality, and perhaps in sweetness, through this unfortunate lack of physical health. It is all very well to talk of delicate persons who are the light and life of the household, to whom everyone turns for counsel and for sympathy. They are more often to be found in books than in real life; and when you do come across them, they are nearly always persons who have had to give up the struggle, and are "laid aside," a little apart from the burdens and toils of everyday life for which they have at last been pronounced to be unfit. It is a very different thing to take one's part in life, and go about very much as other people do, with this miserable sense of unfitness ever pressing one down, and taking the zest out of everything. Those who have to do it know very well that brightness is the great desideratum, if one wishes to be a pleasant entertainer or visitor, an attractive companion, a helpful and ever-welcome friend. But how can they be bright with this constant weight upon their spirits? If they determine not to let it conquer them, they are bright with a ghastly brightness which is too evidently forced to deceive anyone; if they sink back into their natural selves, they are dull, uninteresting, wanting in the energy and vigour, the life and joyousness, which are so pleasantly infectious, and make those who possess them admired, sought after, and be loved.

To rejoice in the love of one's fellow-creatures is no more than natural; and it is natural also that the sense that one is less to them than one might be should cause real suffering. It seems to us sometimes that persons who are out of health actually shrink from us, and seem to prefer being left to themselves as much as possible. And so, perhaps, they may; for the lassitude and dulness of spirits which so often oppresses them makes them unable to rouse themselves, conscious that they are irritable, out of harmony with our feelings, unfit to respond to demands upon their spirits, their thoughts, perhaps even their affections. But do not suppose that, while seeming to shrink from intercourse with you, they do not feel how much they are missing; do not judge them incapable of appreciating popularity, admiration, and love, because they seem to have lost the power of attracting these to themselves.

